# Bimetallic Catalytic Systems Based on Sb, Ge and Ti for the Synthesis of Poly(ethylene terephthalate-*co*-isosorbide terephthalate)

**DOI:** 10.3390/polym9110590

**Published:** 2017-11-09

**Authors:** Nicholas Stanley, Thomas Chenal, Thierry Delaunay, René Saint-Loup, Nicolas Jacquel, Philippe Zinck

**Affiliations:** 1 University Lille, CNRS, Centrale Lille, ENSCL, Univ. Artois, UMR 8181—UCCS—Unité de Catalyse et Chimie du Solide, F-59000 Lille, France; n.stanley@hotmail.co.uk or nicholas.stanley@ifmas.eu (N.S.); thomas.chenal@univ-lille1.fr (T.C.); 2 IFMAS, Institut Français des Matériaux Agro-Sourcés, 60 Avenue du Halley, F-59650 Villeneuve-d’Ascq, France; thierry.delaunay@ifmas.eu; 3 Roquette Frères, 62080 Lestrem CEDEX, France; rene.saint-loup@roquette.com (R.S.-L.); nicolas.jacquel@roquette.com (N.J.)

**Keywords:** isosorbide, poly(ethylene terephthalate), catalysis

## Abstract

The insertion of rigid monomers such as isosorbide into poly(ethylene terephthalate) (PET) allows for the access of polymers with improved properties, notably in terms of thermal stability. This biobased monomer is however poorly reactive, and harsh reaction conditions lead to color concerns regarding the resulting polymer. This has motivated the development of catalytic systems enabling an increase of the reaction rate and a good coloration. In this study, we have assessed bimetallic catalytic systems based on the main metals used for PET catalysis, i.e., antimony, germanium and titanium, for the synthesis of poly(ethylene terephthalate-*co*-isosorbide terephthalate) (PEIT). The Sb_2_O_3_/Ti(OiPr)_4_ combination leads to a high reaction rate while maintaining an acceptable coloration. On the other hand, combining Sb_2_O_3_ with GeO_2_ affords the formation of poly(ethylene terephthalate-*co*-isosorbide terephthalate) without coloration concerns and a reaction rate higher than that observed using the single metal catalysts. Molecular weights and microstructure including diethyleneglycol (DEG) and isosorbide contents are also discussed, together with the thermal properties of the resulting PEIT. The GeO_2_/Ti(OiPr)_4_ is also assessed, and leads to average performances.

## 1. Introduction

**Scheme 1:**
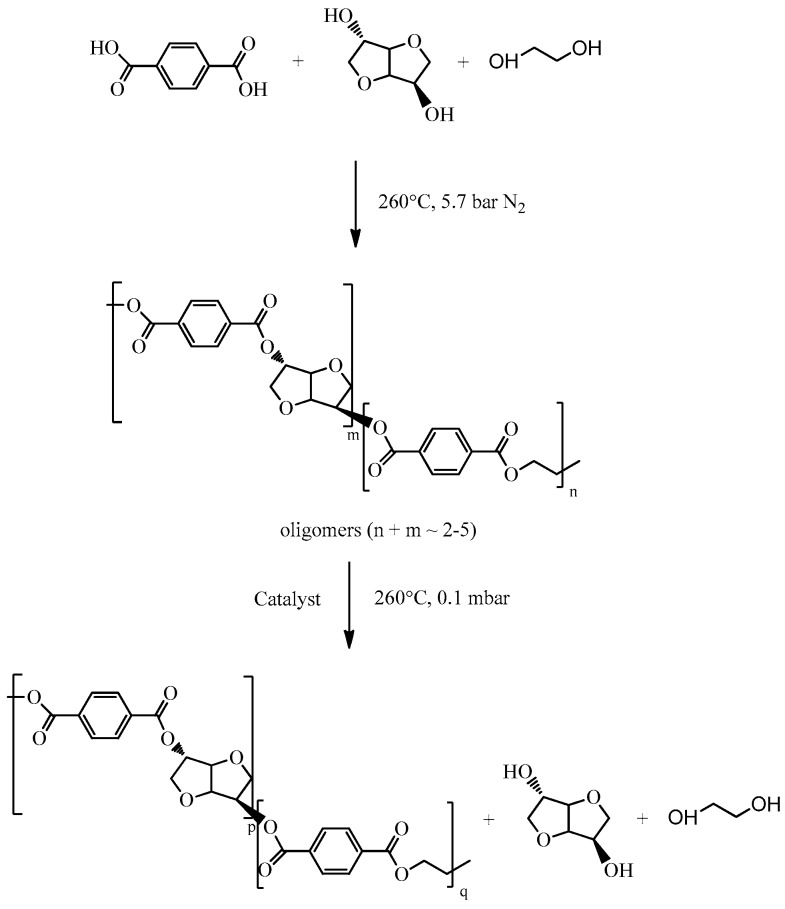
Esterification and transesterification steps.

Poly(ethylene terephthalate) (PET) is one of the major polymers produced worldwide. Due to its good mechanical, thermal and barrier properties, this semi-crystalline thermoplastic is notably used for liquid packaging. The insertion of rigid monomers such as isosorbide (represented in Scheme 1) into the macromolecular structure of PET enables a substantial increase in the glass transition temperature (*T*_g_) of the polymer [1,2,3,4,5,6], which can open the way to new applications such as hot-filling, for example. Isosorbide is a biobased, harmless diol monomer belonging to the family of 1,4:3,6-dianhydrohexitols. It is produced industrially via dehydration of sorbitol, the latter being obtained by hydrogenation of glucose. Isosorbide is also a good candidate for the replacement of bisphenol A, which is toxic, and has also gained high importance in recent years for the production of novel materials for food packaging applications, like furanoate polyesters [7,8,9,10]. An important drawback must however be tackled when considering its insertion into PET. The two hydroxyl functions of isosorbide are secondary, which renders the monomer poorly reactive. Catalytic systems enabling a fast reaction must thus be developed.

Most of the catalytic systems currently developed and used for the synthesis of poly(ethylene terephthalate) are based on three metals: antimony, germanium and titanium [11,12]. Titanium catalysts, usually in the form of titanium alkoxides, are known to be the most reactive catalysts in terms of chain-growth for the polymer, but also in terms of degradation [13], leading to polymers with high coloration. Antimony compounds are known to provide a good balance between reactivity and selectivity, meaning that the reaction times are relatively short and the final products have acceptable coloration, any coloration being attributed to the precipitation of the antimony metal. Sb catalysts are the most commonly used catalysts. Another group of catalysts that have shown a lot of potential are germanium-based compounds. They are known for leading to PET with unique properties in terms of the final products’ color, molecular weight and hydrolytic stability. They are, however, rather expensive and thus less used.

The success of Sb, Ge and Ti based catalysts for the synthesis of PET has motivated their assessment for the synthesis of poly(ethylene terephthalate-*co*-isosorbide terephthalate) (PEIT), as reported in the patent literature. The presence of isosorbide in the reactive medium was found to lead to significant decrease of the reaction rate compared to PET synthesis when using antimony [14] and germanium [15,16] oxide as catalysts. Titanium alkoxides as well as TiO_2_/SiO_2_ combinations were also assessed for the synthesis of PEIT, leading to higher coloration of the final polymer than Sb and Ge based catalysts [17]. Substantial improvements of the synthesis of PEIT were obtained by combining antimony with a main group metal such as Li, Mg and Al [18], or by using a bimetallic Ge/Al system [19]. Synergistic effects were also reported using a combination of antimony with a heterogeneous aluminum based *co*-catalyst for the synthesis of poly(ethylene terephthalate-*co*-ethylene isophthalate) [20]. Alternative strategies combining transesterification, cyclization and polycondensation and leading to isosorbide terephthalate based poly(ester-*co*-carbonate) copolymers were also proposed in the literature [21]. To our knowledge, bimetallic combinations involving the main PET catalysis metals have never been assessed for the synthesis of PEIT. We report herein the use of Sb/Ti, Sb/Ge and Ti/Ge bimetallic catalytic systems for this purpose. We provide also a comparison of the performances of the single metal catalysts in terms of kinetics, microstructure, molecular weights and color of the polymer obtained, which is also lacking in the current literature as far as we know.

## 2. Materials and Methods

### 2.1. Materials

Terephthalic acid and antimony oxide were supplied by Acros (Geel, Belgium). Ethylene glycol, germanium oxide and titanium tetraisopropoxyde (Tyzor TPT) were supplied by Sigma Aldrich (St. Louis, MO, USA). Isosorbide commercialized under the trade name Polysorb P was supplied by Roquette Frères (Lestrem, France). Irgamod 195, a calcium phosphonate stabilizer used as antioxidant was kindly supplied by BASF (Ludwigshafen, Germany).

### 2.2. Oligomer Synthesis

Ethylene glycol (1041.9 g, 16.8 mol), isosorbide (350.5 g, 2.4 mol), terephthalic acid (2656.1 g, 16 mol), sodium acetate (0.181 g) and Irgamod 195 (0.35 g) are introduced into a 7.5 L stainless-steel batch reactor equipped with a heating system, a mechanical stirrer with torque measurement, a distillation column, a vacuum line and a nitrogen-gas inlet. The system is placed under inert gas via 4 cycles of vacuum/nitrogen gas at between 60 and 80 °C. The reaction medium is then heated to 260 °C under 5.7 bars of pressure whilst under constant stirring at 150 rpm. The rate of esterification is estimated by the quantity of distillate collected. Once the esterification is finished, the oligomers are recovered, cooled and ground.

### 2.3. Polymer Synthesis

PEIT oligomers synthetized in the previous step (40 g) are introduced into a glass reactor and the system is sealed. The apparatus is equipped with a heating system, a mechanical stirrer with torque measurement, a vacuum line and a nitrogen-gas inlet. The reactor is placed under inert gas via three cycles of vacuum/argon and heated to 240 °C to melt the oligomers. Whilst performing the cycles of vacuum/argon, the catalyst is placed in ethylene glycol (1 mL) and stirred under argon to form the catalytic solution. Once the oligomers have melted, the catalytic solution is added to the system. At this point, the reactor is simultaneously heated to 260 °C whilst stirring at 50 rpm is applied. When applying the stirring, the viscosity of the reaction medium begins to be measured on a computer using the program “labworld soft (IKA^®^ Works, Inc., Wilmington, NC, USA)”. The reactor is then slowly placed under vacuum during approximately 40 min, reaching pressures of less than 0.1 mbar. The reaction can begin at less than 0.5 mbar which is normally reached at 30 min. The reaction is considered finished when the viscosity no longer increases or reaches 70 N·cm. Once the reaction is finished, the polymer is recovered and placed on a metal surface to cool.

### 2.4. Analyses

Viscosimetry analyses were performed using an automated Ubbelohde capillary system at 35 °C. Samples were prepared at concentrations of 5 g/L in 2-chlorophenol, heated at 135 °C for 2 h with stirring to aid with dissolution before filtration. The reduced viscosity of the samples was calculated using the following equation:

(1) $$\eta_{\mathrm{red}}=\frac{t-t_{s}}{t_{s}\cdot C}$$

where *t* is the time of the analysis for the sample, *t*_s_ is the time of the analysis for the pure solvent and *C* is the concentration of the sample.

Size exclusion chromatography was performed using a mixture of chloroform and 1,1,1,3,3,3 hexafluoro-2-propanol (95:5 vol %) on Agilent Technologies 1260 Infinity (Agilent Tech-nologies Inc., Santa Clara, CA, USA) with Shodex column chromatography (K-G, K804 and K802.5). Samples with a concentration of 5 g/L were injected under a flow rate 1 mL/min. Signals were detected using a refractive index (RI) detector calibrated using PMMA standards (3070, 7360, 18,500, 68,800 and 211,000 g/mol).

DSC analyses were performed under nitrogen atmosphere using a Q20 TA instrument (New Castle, DE, USA), using the following cycles: cool down to 0 °C at 20 °C/min, heat to 280 °C at 20 °C/min (first heat), cool down to 0 °C at 20 °C/min, heat up to 280 °C at 20 °C/min (second heat).

^1^H NMR spectra of the polymers were recorded on a Bruker Avance 300 MHz instrument (Bruker, Billerica, MA, USA) at 300 K using a mixture of deuterated chloroform and deuterated trifluoroacetic acid (3:1 vol %). The chemical shifts were calibrated using the residual resonances of the solvent.

Colorimetry analyses were performed using an Agilent Cary 60 spectrophotometer (Agilent Tech-nologies Inc., Santa Clara, CA, USA). All samples were prepared at concentrations of 50 g/L using a mixture of chloroform and 1,1,1,3,3,3 hexafluoro-2-propanol (95:5 vol %). Values were measured using a standard illuminant *C* light source at 2 degrees. The yellowness index YI was calculated using the following equation:

(2) $$YI=\frac{100\left(C_{x}X-C_{z}X\right)}{Y}$$

where *X*, *Y* and *Z* are the CIE tristimulus values obtained during analyses. *C*_x_ and *C*_z_ are coefficients depending on the illuminant and observer used. For this study, *C*_x_ = 1.2769 and *C*_z_ = 1.0592.

## 3. Results and Discussion

**Figure 1:**
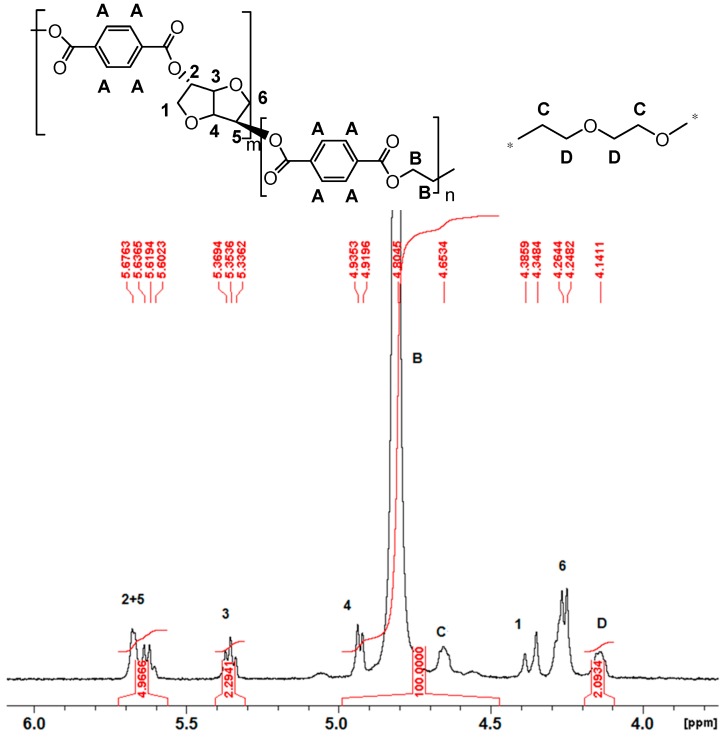
NMR spectra of a typical poly(ethylene terephthalate-*co*-isosorbide terephthalate) PEIT (entry 2) in the 4–6 ppm zone and assignments.

We choose to assess the influence of the catalyst on the transesterification step of the reaction represented in Scheme 1, bottom. A batch of oligomers was synthesized according to the procedure reported in the experimental part and used as starting material. The reaction was stopped either (i) after 3 h or (ii) when reaching a constant viscosity or (iii) when reaching a predefined maximal viscosity corresponding to a torque of 70 N·cm for our experimental set-up. The ^1^H NMR spectrum of a typical poly(ethylene terephthalate-*co*-isosorbide terephthalate) is presented in Figure 1.

The signals characteristic of isosorbide can be seen in addition to the main PET signals, allowing determination of the percentage of isosorbide from the protons **2** and **5**. Small amount of diethylene glycol units in the polymer can also be noticed and determined from the **D** protons. These units, which are present in small quantities, results from the etherification of two ethylene glycol units. They bring flexibility to the resulting material and tend to decrease its glass transition temperature [22]. The method used for the determination of the percentage of isosorbide and DEG is detailed in Appendix A.

**Figure 2:**
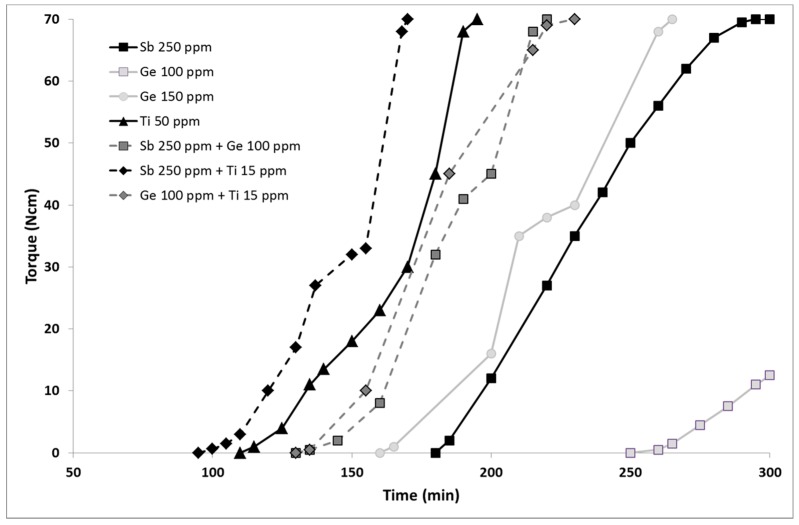
Evolution of the torque as a function of the reaction time.

Results of the synthesis of poly(ethylene terephthalate-*co*-isosorbide terephthalate) starting from the oligomers and using bimetallic combinations are presented in Table 1 in comparison with the single metal catalysts, which were also determined in this study. The catalyst quantity is fixed on the basis of PET catalysis (classical conditions: Ti 8 ppm, Ge 80 ppm and Sb 250 ppm), a little higher in some cases as isosorbide is expected to react slowly. Sb_2_O_3_ (entry 2) leads to a number-average molecular weight around 34,000 g/mol with a dispersity around 2 within our experimental conditions. These values can be compared to an experiment conducted without catalyst and stopped at 300 min (entry 1). The microstructure is composed of ca. 9% isosorbide together with DEG content at ca. 2%. A titanium alkoxide was then assessed (entry 3). The number-average molecular weight and η_red_ of the polymer obtained using 50 ppm titanium tetraisopropoxyde are higher than those obtained using 250 ppm Sb_2_O_3_ (entry 2). The thermal properties of the polymers are given in Table 2. DSC analyses show that the glass transition temperature (*T*_g_) of the sample obtained using the Ti catalyst is close to, but still smaller than that of the PEIT synthetized using Sb_2_O_3_. This might be linked partially to a slightly lower content of isosorbide, around 8%. Experiments performed using germanium oxide as catalysts show that 150 ppm of GeO_2_ (entries 4–5) are required to reach polymer properties similar to those of entries 2 and 3. If the nature of the metal does not significantly influence the amount of diethylene glycol units in the resulting PEIT, Sb leads to the highest isosorbide content, ca. 1% higher than Ge and Ti. In terms of colouration, the titanium catalyst leads to the highest yellowness index, likely due to an increase in degradation commonly observed when using such catalysts. The increase in yellowing indicates degradation that could also explain in part the decrease in *T*_g_ values observed. We were further interested in the kinetics of these transesterification reactions. The evolution of the torque in the course of the reactions represented in Figure 2 shows that the reaction is significantly faster using the titanium catalyst, with an interesting *t*_0_ value (time corresponding to the apparition of the torque curve in Figure 2) 50 to 75 min lower than that observed for germanium and antimony, respectively.

**Figure 3:**
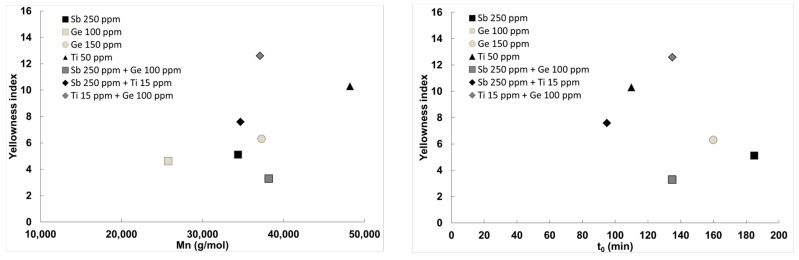
Yellowness index as a function of the number-average molecular weight (**left**) and t_0_ (**right**).

Bimetallic systems were then assessed. The Sb/Ge combination leads to a higher reaction rate than that observed using the metals alone (see Figure 3 and entry 6 vs. 4 and 2). Similar molecular weights and thermal data are obtained, with intermediate isosorbide incorporation and a lower diethylene unit content. The most important improvement is the coloration with a yellowing index lower than that obtained using the metals alone. A significant synergy can thus be noticed between these two metals, resulting in the best yellowness index/molecular weight combination of this study. This is presented in Figure 3, left. When antimony is combined to 15 ppm titanium, a very fast reaction is observed, with a *t*_0_ of 95 min, resulting in the best yellowing index/*t*_0_ combination (see Figure 3, right). The coloration remains indeed acceptable, yet still higher than that of antimony alone, with a yellowing index of 7.3. Other polymer characteristics are similar to those obtained using the Sb/Ge combination. The Ge/Ti combination was finally assessed. If rather high molecular weights are obtained, this combination leads to coloration concerns with a yellowing index higher than 12 and a glass transition temperature of 86 °C. The kinetic is similar to the Sb/Ge but lower to the Sb/Ti. Among the three combinations, a good match is thus observed when combining antimony with titanium or with germanium, while a mismatch effect is observed between Ge and Ti, as seen in entry 3.

The influence of the nature of the metal has been tentatively rationalized for PET on the basis of the measurement of rate parameters for polymerization and thermal degradation for a model system based on bis(2-hydroxyethyl) terephthalate [13]. It can be seen that that Ti based catalysts exhibit a higher polymerization rate than Sb based catalysts, but also a higher degradation rate. Ge was not studied in this work, but lies probably in between Ti and Sb in terms of activity. Titanate catalysts are also known to induce degradation in polyesters under certain conditions [23]. This can explain the results observed on single metal catalysts, and also the increased yellowness indexes observed in the bimetallic combinations containing Ti. The decrease in coloration observed using the Sb/Ge combination may be explained tentatively as follows. The coloration using Sb catalysts is generally attributed to the precipitation of antimony metal. The presence of germanium may induce a stabilization of antimony at the oxidation state III, leading to a reduction of the coloration. The formation of a bimetallic species may eventually be advanced.

## 4. Conclusions

Sb_2_O_3_, GeO_2_, Ti(OiPr)_4_ and their combinations were assessed as catalysts for the synthesis of poly(ethylene terephthalate-*co*-isosorbide terephthalate). Sb based catalysts lead to the highest isosorbide incorporation, an acceptable coloration but the lower reactivity. Ti systems lead on the other hand to the fastest reactions, but also the highest coloration. 150 ppm GeO_2_ allows a slightly faster reaction than 250 ppm Sb with similar coloration, but lower isosorbide incorporation. The addition of 15 ppm Ti(OiPr)_4_ to Sb and Ge systems leads to a significant increase of the reaction rate, without significant alterations of the polymer properties for Sb *co*-catalysis, but to a higher coloration/degradation for the germanium based system. The Sb/Ti combination can thus be considered a good candidate when the reaction rate is to be optimized. The combination of Sb_2_O_3_ with GeO_2_ was further assessed. It affords to make a PEIT with the lowest coloration of the whole study. This synergistic effect is also accompanied by an increase of the reaction rate, although less impressive than Ti based systems. This combination should thus be preferred if the colour is a priority. It is shown in this study that using the right combination between catalysts classically used for PET synthesis, such Sb_2_O_3_ and GeO_2_ and titanium alkoxides, allows one to tune the performances of catalytic systems for PEIT by favouring either a good colour or a high reactivity, with the unfavoured one being kept at an acceptable level.

## Figures and Tables

**Table 1:** Kinetic results, microstructure, reduced viscosity and molecular weights of PEIT formed using the different catalysts.

Entry	Catalyst	Amount (Metal wt. ppm)	*t*_0_^1^ (min)	Isb ^2^ (mol %)	DEG ^3^ (mol %)	η_red_^4^ (mL/g)	*M*_n_^5^ (g/mol)	*Ð*_m_^5^
1	-	-	-	8.2	4.9	27.3	10,800	2.07
2	Sb_2_O_3_	250	185	9.2	1.9	102.7	34,400	2.07
3	Ti(O^i^Pr)_4_	50	110	8.2	1.6	115	48,200	2.07
4	GeO_2_	100	250	8.0	1.5	68.6	25,800	1.97
5	GeO_2_	150	160	8.1	1.4	105.6	37,300	2.21
6	Sb_2_O_3_/GeO_2_	250 100	135	8.4	1.4	105.5	38,200	2.19
7	Sb_2_O_3_/Ti(O^i^Pr)_4_	250 15	95	8.5	1.5	104.5	34,700	2.11
8	GeO_2_/Ti(O^i^Pr)_4_	100 15	135	8.2	1.2	118.9	49,000	2.06

^1^ Time corresponding to the apparition of the torque curve in Figure 3; ^2^ isosorbide content measured by ^1^H NMR; ^3^ diethylene glycol content measured by ^1^H NMR; ^4^ reduced viscosity; ^5^ number average molecular weight and dispersity measured by size exclusion chromatography in a 95/5 chloroform/1,1,1,3,3,3 hexafluoro-2-propanol mixture using PMMA standards.

**Table 2:** Thermal properties and colorimetric analyses of PEIT formed using the different catalysts.

Entry	Catalyst	Amount (Metal wt ppm)	*T*_g_^1^ (°C)	*T*_m_^1^ (°C)	L* ^2^	a* ^3^	b* ^4^	YI ^5^
1	-	-	83	230	97.9	−0.1	3.7	6.2
2	Sb_2_O_3_	250	91	227	98.4	−1.4	3.3	5.1
3	Ti(O^i^Pr)_4_	50	88	229	97.6	−1.9	6.3	10.3
4	GeO_2_	100	81	230	98.6	−1.2	2.9	4.6
5	GeO_2_	150	88	227	97.1	−1.5	3.9	6.3
6	Sb_2_O_3_/GeO_2_	250 100	89	227	78.2	−0.9	1.8	3.3
7	Sb_2_O_3_/Ti(O^i^Pr)_4_	250 15	90	228	97.5	−1.6	4.7	7.6
8	GeO_2_/Ti(O^i^Pr)_4_	100 15	86	227	95.7	−2.0	7.5	12.6

^1^ Glass transition and melting temperatures measured by DSC; ^2^ Whiteness of the polymer. ^3^ Colour of the polymer in the range of green (negative values) to red (positive values); ^4^ Colour of the polymer in the range of blue (negative values) to yellow (positive values); ^5^ Yellowness index (see experimental part).

## Appendix A

From protons 2 and 5: Isb(4H) = 2 × 4.97 = 9.94
From protons D: DEG(4H) = 2.09
From protons 4,B and C: EG(4H) + Isb(1H) + DEG(4H) = 100
Isb(1H) = Isb(4H)/4 = 2.485
Thus EG(4H) = 100 − 2.09 − 2.485 = 95.425
EG(4H) + Isb(4H) + DEG(4H) = 107,455
Isb (%) = 9.94/107.455 × 100 = 9.2
DEG (%) = 2.09/107.455 = 1.9
